# Behavioral, Functional Imaging, and Neurophysiological Outcomes of Transcranial Direct Current Stimulation and Speech-Language Therapy in an Individual with Aphasia

**DOI:** 10.3390/brainsci14070714

**Published:** 2024-07-16

**Authors:** Sameer A. Ashaie, Julio C. Hernandez-Pavon, Evan Houldin, Leora R. Cherney

**Affiliations:** 1 Think and Speak, Shirley Ryan AbilityLab, Chicago, IL 60611, USA; sashaie@sralab.org (S.A.A.); ehouldin@sralab.org (E.H.); 2 Physical Medicine and Rehabilitation, Feinberg School of Medicine, Northwestern University, Chicago, IL 60611, USA; 3 Department of Psychological Sciences, Kansas State University, Manhattan, KS 66506, USA; juliohpavon@ksu.edu

**Keywords:** aphasia, tDCS, fMRI, EEG, scripting, conversation, cathodal, speech-language therapy, functional connectivity

## Abstract

Speech-language therapy (SLT) is the most effective technique to improve language performance in persons with aphasia. However, residual language impairments remain even after intensive SLT. Recent studies suggest that combining transcranial direct current stimulation (tDCS) with SLT may improve language performance in persons with aphasia. However, our understanding of how tDCS and SLT impact brain and behavioral relation in aphasia is poorly understood. We investigated the impact of tDCS and SLT on a behavioral measure of scripted conversation and on functional connectivity assessed with multiple methods, both resting-state functional magnetic resonance imaging (rs–fMRI) and resting-state electroencephalography (rs–EEG). An individual with aphasia received 15 sessions of 20-min cathodal tDCS to the right angular gyrus concurrent with 40 min of SLT. Performance during scripted conversation was measured three times at baseline, twice immediately post-treatment, and at 4- and 8-weeks post-treatment. rs–fMRI was measured pre-and post-3-weeks of treatment. rs–EEG was measured on treatment days 1, 5, 10, and 15. Results show that both communication performance and left hemisphere functional connectivity may improve after concurrent tDCS and SLT. Results are in line with aphasia models of language recovery that posit a beneficial role of left hemisphere perilesional areas in language recovery.

## 1. Introduction

Aphasia is an acquired multi-modality disturbance of language that typically occurs after a left-hemisphere stroke. It impacts spoken language comprehension and production, reading, and writing. The disorder can also result in depression, decreased quality of life, and social isolation [1,2,3]. While speech-language therapy (SLT) is the gold-standard in treating persons with aphasia, its long-term effectiveness has not been established and residual language impairments remain even after intensive SLT [4]. Due to recent advances in our understanding of aphasia and brain–behavior relations, novel brain stimulation approaches to treatment are emerging. The neurological basis for these interventions is long-lasting functional and structural plasticity of both intact perilesional areas and cortical areas remote from the injury [5,6]. The neuroplastic nature of the brain makes it ideal for cortical stimulation (e.g., transcranial direct current stimulation (tDCS) and transcranial magnetic stimulation) since cortical stimulation can directly boost underlying plasticity to supplement the rehabilitation process from SLT [7,8].

Recent research suggests that language recovery in persons with aphasia can be improved by pairing SLT with a safe and low-cost form of brain stimulation known as tDCS [9]. tDCS does not induce neuronal firing but modulates the resting membrane potential of the neurons, changing their threshold and impacting the ease and rate at which neurons fire [10]. It is assumed that tDCS effects are polarity specific such that anodal stimulation increases cortical excitability and improves performance, while cathodal stimulation decreases cortical excitability and worsens performance [10,11]. The choice of whether to use anodal or cathodal stimulation in aphasia is motivated by models of aphasia recovery [12]. These models suggest that good recovery is based on successful recruitment of the left-hemisphere perilesional areas. However, some studies have found the contra-lesional right-hemisphere to have both a maladaptive and a facilitatory role [13,14,15,16]. For example, Keser et al. [16] found that greater reliance on the right hemisphere homologues of language tracts may have a negative impact on language recovery while other studies (e.g., Turkeltaub et al. [13]) have found that the right hemisphere may aid in language recovery. These models of language recovery in turn have informed how tDCS is used to aid language recovery in persons with aphasia. Indeed, a recent scoping review of non-invasive brain stimulation studies found that most studies used anodal tDCS to increase cortical excitability of the left perilesional areas [17]. However, a few studies have also used cathodal tDCS to target the right-hemisphere areas to suppress the maladaptive nature of the right-hemisphere, so that left perilesional areas could be more optimally recruited for language recovery [12,18]. 

The majority of tDCS studies in aphasia have used behavioral outcomes as their primary measure, with naming being the most common outcome measure [9,18,19,20,21]. For example, Fridriksson et al. [22] found that naming improves after anodal left-hemisphere tDCS in persons with aphasia. Only a few studies [23,24] have investigated the impact of tDCS on conversational abilities. Indeed, a recent meta-analysis by Elsner et al. [9] found that 112 patients in 3 studies received active tDCS to improve functional communication while 298 patients in 11 studies received active tDCS to improve naming. However, the meta-analyses of tDCS and aphasia by Elsner et al. [9] as well as a meta-analysis by You et al. [20] indicated that the impact of tDCS on functional communication in persons with aphasia is inconclusive. Therefore, the need to explore how tDCS and SLT impact functional communication remains. A few studies (n = 9/36 [18]) have also used functional magnetic resonance imaging (fMRI) to understand cortical changes induced by tDCS and SLT. Some of these studies found changes in inter-hemispheric connectivity, while others found changes in intra-hemispheric connectivity [25,26]. However, the majority of the fMRI studies used naming as both the treatment and outcome measure, with the fMRI as a tool for determining electrode placement [18]. Therefore, our understanding of how tDCS in combination with language treatments impacts cortical activity to induce language recovery in the context of functional communication, such as conversation, is still unclear [12,17]. It is possible that tDCS induced cortical changes are dependent on the type of stimulation (e.g., anodal vs. cathodal), the tDCS cortical target (e.g., Broca’s vs. angular gyrus), and the type of language therapy (e.g., naming vs. scripting) provided. Indeed, variability in behavioral outcomes in persons with aphasia can be attributed to our lack of understanding of how tDCS combined with different types of SLT modulates residual brain networks to impact behavioral outcomes [9,27]. 

In the present study, we investigated changes in scripted conversational performance and functional connectivity after 15 days of tDCS and SLT in an individual with chronic aphasia. Changes in functional connectivity were investigated using both resting-state fMRI (rs–fMRI) and resting-state electroencephalography (rs–EEG) to provide converging evidence on the impact of concurrent tDCS and SLT on residual brain connectivity. Understanding how tDCS modulates residual brain networks to improve behavioral performance will allow us to devise larger studies that can maximize the potential of tDCS as an adjuvant to SLT.

## 2. Method

### 2.1. Participant

**Figure 1:**
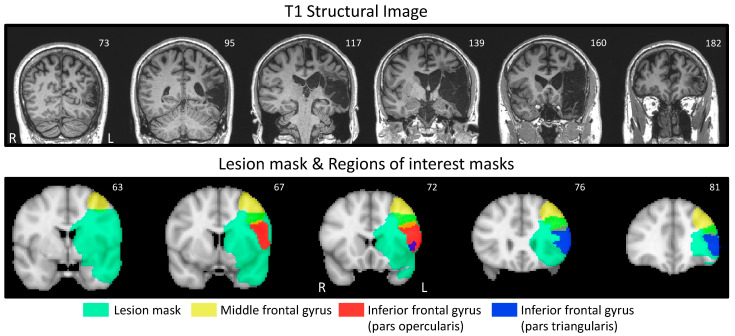
(**Top**) Coronal views of T1 structural image, in native space (slice number indicated in top right corner of each slice), presented in radiological view. (**Bottom**) Coronal views of regions of interest (ROI) masks overlapping with P1’s lesion mask (aquamarine underlay). ROI: middle frontal gyrus (MFG, yellow), inferior frontal gyrus (IFG, pars opercularis; orange) and IFG, pars triangularis (blue). ROI masks are from the Harvard–Oxford Cortical Structural Atlas, at 75% threshold (mask colors also have reduced opacity, so that overlap can be seen; e.g., green indicates MFG overlap with lesion mask). All masks are in MNI152 space, overlaid on the MNI152 standard brain (slice number indicated in top right corner of each slice), and are presented in radiological view. Please note that the slice numbering for the top image reflects the higher resolution (i.e., greater total number of slices) of the T1 structural image, as compared to the fewer total number of slices for the MNI image. Further, the slices in the top image were selected to capture the full extent of the lesion, whereas the lower image slices were selected to illustrate the overlap of the lesion with the ROI masks.

**Figure 2:**
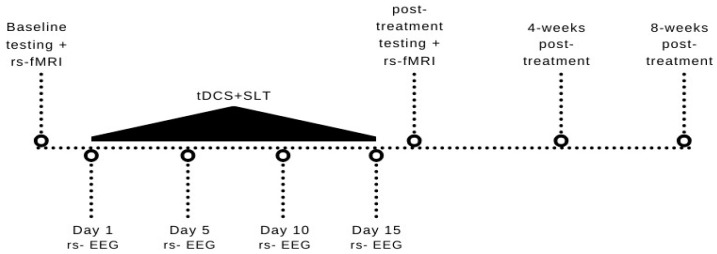
Study timeline. tDCS and SLT were administered for 15 days. Baseline language testing and rs–fMRI were collected immediately pre- and post-treatment. rs–EEG was collected on day 1, 5, 10, and 15 of the treatment. Language testing also occurred 4 and 8-weeks post-treatment.

The participant P1 was a 49-year-old right-handed male at the time of this study. He suffered a middle cerebral artery ischemic stroke 70 months before the onset of this study. The participant was administered a variety of assessments immediately prior to the start of the present study. P1’s Aphasia Quotient (AQ) on the Western Aphasia Battery-Revised (WAB-R [28]) was 65.8. His naming accuracy on the Philadelphia Naming Test-Short Form (PNT [29]) was 70%. He also scored 34/40 on the non-verbal Arizona Semantic Test, indicating that his semantic knowledge was relatively unimpaired. We also investigated his communication confidence using the Communication Confidence Rating Scale for Aphasia (CCRSA [30,31]), in which he scored 30/40. Communication confidence has been shown to be independent of aphasia severity and a predictor of overall quality of life for persons with aphasia [32]. The CCRSA is psychometrically valid and has been used in a variety of settings to measure communication confidence in persons with different levels of aphasia severity [30,31]. P1 did not meet the threshold for depression on the Center for Epidemiologic Scale- Revised (2/60) [33] and indicated no deficits in quality of life on the Stroke and Aphasia Quality of Life Scale-39 (4.77/5) [34]. Table 1 provides the breakdown of P1’s WAB-R subtest scores. Notably, P1’s lesion (Figure 1) strongly overlapped with regions of interest (ROI) associated with speech planning [35], i.e., left-lateralized caudal inferior frontal cortex (cIFG) and caudal middle frontal gyrus (cMFG). (Please note that Figure 1 indicates the complete MFG and IFG). P1 was recruited from the community and was undergoing no SLT during the duration of the study. Ethical approval was obtained from the Institutional Review Board of Northwestern University. Figure 2 summarizes the timeline of the study.

### 2.2. Speech-Language Therapy

P1 received SLT in the form of computer-based script training. Script training is an intensive standard-of-care-treatment in which the participant repeatedly practices (e.g., pointing, choral reading, reading aloud independently) sentences within different contexts as either a dialogue or a monologue [36,37,38,39]. Script training as a speech and language rehabilitation treatment is used routinely in persons with stroke and primary progressive aphasia [40,41]. The aim of the script training is for persons with aphasia to automatically produce key pieces of the practiced script in daily life communicative situations. Importantly, the script topics practiced in treatment sessions are generally based on personally relevant topics. For example, a patient interested in ordering food in a restaurant will practice a script related to ordering food in a restaurant in both dialogic and monologic forms with the goal that they will be able to use pieces of the learned script in real-life restaurant situations. P1’s script was ten sentences long at a Grade 5 Flesch–Kinkaid reading level and focused on watching a sporting event. The script was personally relevant to our participant. Script training was provided for 40 min a day for 15 sessions over 3 weeks (i.e., 5 days a week)

### 2.3. Transcranial Direct Current Stimulation

2 mA of direct current was delivered using a Soterix 1x1 constant current stimulator (Soterix Medical., New York, NY, USA) via two rubber encased saline soaked sponges. tDCS electrodes were placed using a neuronavigation system (Localite, Bonn, Germany). 

Neuronavigation was used to co-register P1’s heads in 3-D space with their T1 weighted MR image. Neuronavigation allows us to identify cortical regions of interest and then map those to the scalp of the participant. We then placed a 15 cm^2^ cathodal “target” electrode on the right angular gyrus (CP6 on 10–20 internation EEG system for electrodes) and the 25 cm^2^ anodal “return” electrode on the center of the supraorbital region. The electrodes were secured by a custom-built EEG cap (Easycap, Etterschlag, Bavaria, Germany). Neuronavigation was performed at the beginning of each treatment session to ensure that our placement of electrodes was consistent throughout the treatment sessions. tDCS was applied for the first 20 min of the 40-min SLT session. The tDCS stimulation intensity and duration were based on previous tDCS studies [42]. 

### 2.4. Electric Field Modeling

**Figure 3:**
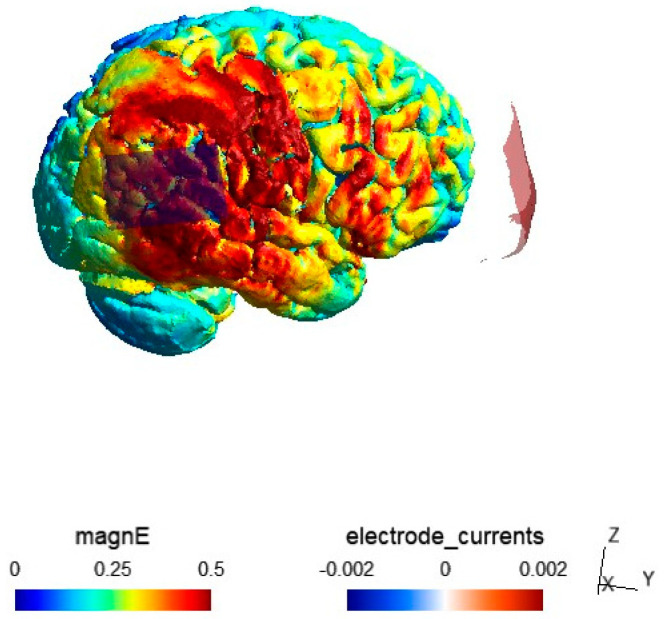
Electric Field modeling of P1’s montage. Current density is strongest in the temporo–parietal and frontal right-hemisphere regions.

Electric field (EF) modeling was performed using SimNIBS 4.0 [43]. SimNIBS is an open-source software that allows simulation of the induced electric field in tDCS. P1’s T1 weighted image was used to generate the tetrahedral head using the charm module, which automatically performs tissue segmentation on the T1-weighted MRI. We also visually checked the segmentation to ensure that charm had segmented the T1-weighted MRI accurately. Electrode sizes were modelled using 15 cm^2^ and 25 cm^2^ sizes with pre-defined SimNIBS conductive values. The simulation results were visualized using the MATLAB R2021b (The Mathworks, Inc., Natick, MA, USA) toolbox gmsh [44]. EF modeling showed the highest current densities in the temporo–parietal and frontal regions of the right hemisphere (Figure 3). 

### 2.5. Outcome Measures

#### 2.5.1. MRI and fMRI Recordings

P1 was scanned pre- and immediately post-treatment in a 3T Siemens Prisma whole body scanner (Siemens, Erlangen, Germany), with echo-planar imaging using the standard radio-frequency head coil. One anatomical image and one resting-state fMRI (rs–fMRI) set of images were acquired both pre- and post-intervention, with the following parameters: high-resolution T1-weighted brain images were collected using integrated parallel imaging techniques (PAT; GRAPPA) representing receiver coil-based data acceleration methods; voxel size 1 × 1 × 1 mm^3^; TR = 2300 ms; TE = 2.4 ms; flip angle = 9°; acceleration factor of 2; in-plane matrix resolution = 256 × 256; slices = 176; field of view = 256 mm. Rs–fMRI images were acquired immediately after anatomical images, with the following parameters; multi-slice T2*-weighted echo-planar images, with a multiband acceleration factor of 8 (8 × 8 simultaneously acquired slices) with interleaved ordering; TR = 0.555 s; TE = 22 ms; flip angle = 47°; in-plane resolution = 104 × 96; number of slices = 64; spatial resolution = 2 mm^3^; number of volumes = 1110. 

#### 2.5.2. MRI Data Preprocessing

A lesion mask was drawn manually for the pre-intervention anatomical image, using the FSLeyes software from FSL [45] v6.0.3, with the lesion mask for the second anatomical image derived by using the pre- to post-intervention T1 image co-registration transform. Anatomical images were then skull stripped using FSL’s BET function, after merging the lesion mask with the anatomical image, and co-registered to MNI space using FSL’s FMRIB Expert Analysis Tool (FEAT). 

#### 2.5.3. Resting-State MRI (rs–fMRI) Preprocessing

The first 20 volumes of each set of Rs–fMRI images were discarded to accommodate magnetic field stabilization. This left a total of 1090 volumes for functional connectivity analyses. FEAT was then used to implement skull-extraction, slice-time correction, and motion correction. Time series for the BOLD signal were filtered with a Butterworth band-pass filter (0.008 Hz < f < 0.1 Hz) and a non-linear spatial filter (using SUSAN tool from FSL; FWHM = 5 mm). Following this, we regressed the six parameters obtained by rigid body correction of head motion, global signal averaged overall voxels of the brain, white matter signal averaged overall voxels of the eroded white matter region, and ventricular signal averaged overall voxels of the eroded ventricle region. Functional volumes indicating extreme motion were also removed using a custom script to implement volume censoring, and bandpass filtering was applied (0.008–0.1 Hz). Finally, volumes were registered to MNI space using FSL’s FNIRT tool [46].

#### 2.5.4. Behavioral Outcomes

The primary behavioral outcome measure was the accuracy of production of the trained script in a scripted conversational setting immediately following the end of treatment. Conversation as an outcome measure is relevant to persons with aphasia since deficits in conversational abilities can have negative consequences on their overall quality of life [3]. However, there is no gold standard conversational treatment or outcome measures for persons with aphasia [47]. Therefore, different researchers have used different conversation treatments and outcomes in their studies (see Azios et al. [47] for further details). In our study, we used scripted conversation as an outcome measure. In scripted conversation, a speech-language pathologist says each sentence of the trained script in a conversational style without cues and the person with aphasia responds to each sentence in a conversational style. In other words, both the person with aphasia and the speech-language pathologist take part in a rehearsed conversation. The scripted conversation outcome represents a stimulus generalization task in which the setting (i.e., scripted conversation with a real person) is less similar to the setting used for the treatment (i.e., computer-based training) [48]. The scripted conversation was recorded three times at baseline and twice immediately after the last treatment session [49]. Secondary behavioral outcome measures were changes in accuracy of the scripted conversation production at 4- and 8-weeks post treatment to assess maintenance, as well as the WAB-R AQ and scores on the PNT Short Form and the CCRSA. 

#### 2.5.5. Resting-State EEG (rs–EEG) Recordings

Resting-state EEG responses were recorded with eyes open for 3 min before and after tDCS and SLT on days 1, 5, 10, and 15 of treatment. The EEG activity was recorded with a 64-passive electrode system (NeurOne Tesla, Bittium, Kuopio, Finland). The EEG electrodes were placed in an elastic cap according to the International 10–20 System [50]. The reference was placed in the right mastoid. The electro-oculogram (EOG) activity was also recorded for further analysis. The EOG electrodes were placed at the lateral canthus of each eye (horizontal) and above and below the right eye (vertical). The impedances of all electrodes were kept at ≤5 kΩ during the recording sessions. The EEG signals were acquired at 1 kHz in DC mode. The recordings were performed inside a two-layer electrically shielded room (Gaven Industries Inc., Saxonburg, PA, USA). The participant was asked to look at a fixation cross in front of him during the EEG recording to avoid distractions. 

#### 2.5.6. EEG Preprocessing

The EEG data were analyzed offline with MATLAB R2021b (The Mathworks, Inc., Natick, MA, USA) and the MNE processing stream [51]. First, the data were detrended. Then, the bad channels and data segments containing artifacts (for instance, noise, amplitudes exceeding ±30 µV, or strong muscle activity from eye blinks, scalp, or neck muscles) were identified by visual inspection and removed. 

#### 2.5.7. Neuronavigation and Coordinate System Alignment between MRI, EEG, and tDCS

After the EEG was prepared, the electrodes’ locations, three landmarks (nasion, left and right preauricular), and about 200 additional scalp surface points were acquired with a neuronavigation system (Localite, Bonn, Germany). Then, the landmark points were identified from the structural MRI to perform an initial alignment of the coordinate system [51].

## 3. Analysis

### 3.1. Behavioral Data Analysis

Words related to the trained script were scored on a 6-point scale with 0—no response, 1—unintelligible or unrelated response, 2—semantic or phonological paraphasias, 3—minor errors (e.g., missing grammatical morphemes), 4—accurate but delayed or self-corrected response, and 5—accurate and immediate response. Percent accuracy of the maximum score (i.e., 5 × number of script-related words) was calculated. This scoring is based on the NORLA-6 [52], a valid and reliable, script scoring method that is sensitive to change after SLT. The script was scored by a speech-language pathologist not involved in administering the treatment. 

### 3.2. Resting-State fMRI Analysis

Graph measures were based on a functional network defined by 242 regions distributed across cortical and sub-cortical regions. Each region was defined as a 10 mm diameter sphere centered on coordinates reported in Power et al., 2011 [53]. The Brain Connectivity Toolbox [54] was used to compute a variety of graph measures (e.g., modulatory, betweenness centrality, small Worldness, clustering coefficient), including local and modular changes, (e.g., inter- and intra-hemispheric functional connectivity), before and after treatment. Table 2 summarizes these measures pre- and post-treatment. These properties were compared to 18 age- and sex-matched healthy controls (HC, M = 49.4 years, SD = 2.3 years), with rs–fMRI data taken from the 1000 Functional Connectomes Project [55]. In addition, 95% confidence intervals for the HC graph measures were generated with custom bootstrapping code, using the aforementioned data from the 1000 Functional Connectomes Project. Functional connectivity figures were generated by plotting the top 5% FC edges to indicate general FC trends, using the gplot function in MATLAB R2021b (The Mathworks, Inc., Natick, MA, USA). In a prior study [56], we also found that ~5% connectivity density was an ideal threshold for significant correlations between FC and treatment outcomes; however, in the present study, this threshold was selected with visual clarity as the primary consideration. Seed-based correlation was performed using 3dNetCorr from the AFNI software package, v24.1.11 [57,58] in order to assess functional connectivity with angular gyrus-associated regions, such as the default mode network (DMN).

### 3.3. EEG Analysis

The source analysis of the EEG responses was performed with the MNE processing stream [51]. For the forward solution, a three-layer boundary element model (BEMs, with inner skull, outer skull, and scalp) was extracted from the MRI and decimated to 5120 triangles to be used as the volume conductor. MNEs were computed by combining the anatomical MRI and EEG data [59,60,61]. For the inverse solution, the cortical surface from FreeSurfer was decimated to 4098 vertices per hemisphere, the source space was restricted to cortical grey matter, and the dipole orientations were constrained to be perpendicular to the local curvature of the cortical surface. Then, the averaged time courses were extracted from the inferior frontal and angular gyrus from the left and right hemispheres. 

The EEG signals were preprocessed and analyzed in the source space for the EEG connectivity analysis. The EEG signals were extracted from four regions of interest (left and right inferior frontal gyrus (IFG) and left and right angular gyrus (AG)). First, a spectral analysis was performed for each extracted time course between 0 and 100 Hz by a Fast Fourier transform, and then a coherence analysis was computed. The connectivity analysis was performed with custom scripts in MATLAB R2021b (The Mathworks, Inc., Natick, MA, USA) and the use of the “coh” function from FieldTrip [62]. Broad-band coherence values were obtained by averaging the coherence values over the time courses for the theta (4–7 Hz), alpha (8–12 Hz), and beta (13–30 Hz) frequency bands. In order to assess changes in functional connectivity within the right hemisphere, a coherence analysis was performed between the signals extracted from the right IFG and right AG. To assess connectivity changes in the left hemisphere a coherence analysis was performed between the signals extracted from the left IFG and left AG. 

## 4. Results

### 4.1. Behavioral Results

**Figure 4:**
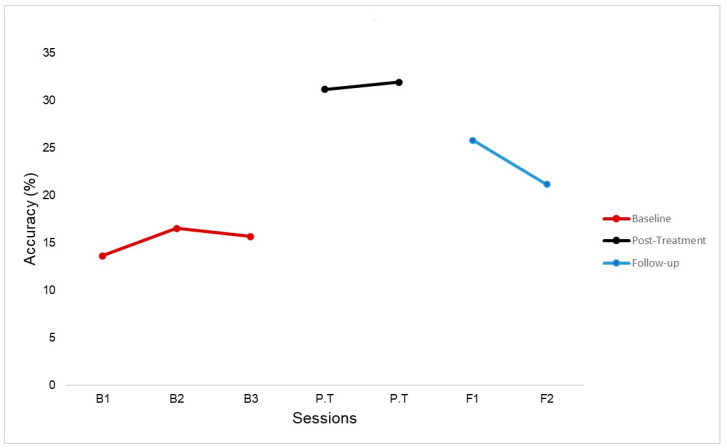
Performance on Scripted Conversation. Percent accuracy on scripted conversation at baseline sessions (B1–B3), immediately post-treatment (P.T) and 4-weeks and 8-weeks follow-up (F1 and F2).

The primary behavioral measure was the change from pre-treatment to post-treatment in the accuracy of the script-related words during a scripted conversation with a speech-language pathologist using the trained script. P1’s performance improved from baseline to immediately post-treatment (106.4% increase) on the scripted conversation (Figure 4). Improvement in the scripted conversation at 4- and 8-weeks post-treatment was also sustained from baseline but decreased immediately from post-treatment (Figure 4). Additionally, P1’s score at 8-weeks post-treatment seemed to trend towards his baseline scores. Further investigation is needed to understand how long improvement in scripted conversation lasts post-treatment. P1’s aphasia quotient on the WAB-R improved by 3 points post treatment (68.7) and by 4 points at 4-weeks and 8 weeks post-treatment (69.5 and 69.9 respectively). There was no improvement in the PNT. P1 also improved by 7 points on communication confidence as measured by the CCRSA immediately post-treatment. 

### 4.2. Resting-State fMRI Results

**Figure 5:**
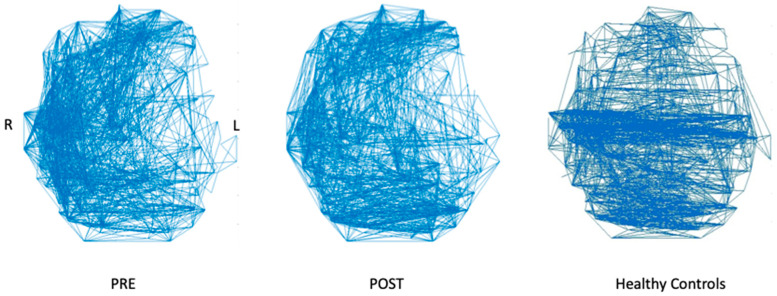
Axial views (radiological convention) of top 5% strongest functional connections for P1 pre-treatment (**left**), P1 post-treatment (**center**) and mean of 18 age- and sex-matched healthy controls (HC, **right**). HC connectivity is dominated by strong bilateral connections. P1’s connectivity shifts noticeably towards stronger bilateral connections, with fewer right-lateralized connections, following treatment, similar to HC.

**Figure 6:**
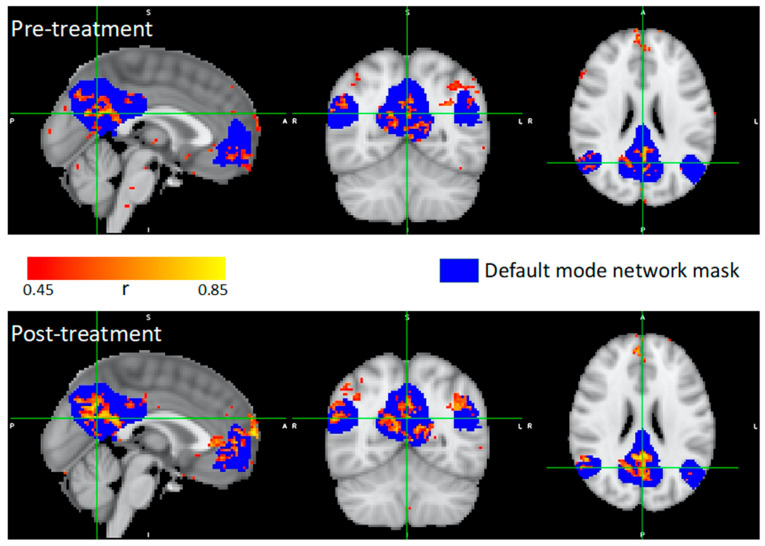
Seed-based correlation analysis (SCA) results, based on P1’s resting-state fMRI data. Mean activity from default mode network (DMN) mask (blue), used as seed. Mask is presented in MNI152 space, overlaid on the MNI152 standard brain. Sagittal, coronal and axial views presented for pre-treatment (**top**) and post-treatment (**bottom**). Correlation values (r) with seed presented, according to color bar. More coherent DMN activity present, following treatment.

Of the resting-state fMRI graph measures, 8/9 were found to change towards those of healthy controls (HC) after treatment (Table 2). In particular, left intra-hemispheric connectivity (left IHC, evaluated as the proportion of 95th-percentile to total-number-of intra-hemispheric FC edges) increased from 0.030 to 0.041, vs. the 0.053 HC mean (95% CI: 0.051–0.056). Concurrently, right IHC decreased from 0.101 to 0.088, vs. the 0.066 HC mean (95% CI: 0.062–0.070), (Figure 5). Fractional anisotropy increased for all white matter tracts of interest (Table 3). Within-DMN FC was also found to increase post-intervention (Figure 6).

### 4.3. EEG Results

**Figure 7:**
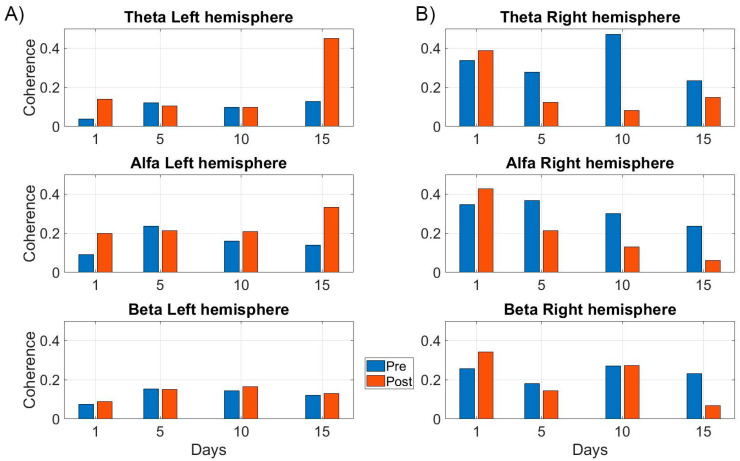
Resting-state EEG connectivity. rs–EEG connectivity results in different EEG frequencies across different treatment sessions. (**A**) depicts the connectivity for the left hemisphere, and (**B**) depicts the connectivity for the right hemisphere.

The EEG connectivity analysis also shows that functional connectivity increased in the left-hemisphere after 15 days of treatment (Figure 7A) for all frequencies (theta, alpha, and beta), whereas functional connectivity decreased in the right hemisphere after the treatment (theta, alpha, and beta) (Figure 7B). To note, we also saw differential changes in EEG connectivity across different tDCS and SLT sessions (Figure 7A,B).

## 5. Discussion

Recent meta-analyses and systematic reviews [9,20,21] have found that the majority of tDCS studies in persons with aphasia used naming as the primary outcome measure. Furthermore, these meta-analyses have found inconsistent effects of tDCS on functional communication in persons with aphasia. These inconsistencies could be attributed to a variety of factors, including inconsistent placement of electrodes and use of different tDCS parameters. For example, some studies used anodal (n = 24) tDCS while other studies used cathodal stimulation (n = 7), which may differentially impact tDCS results [18]. Moreover, Williams et al. [17] found that only 24% of TMS and tDCS studies used neuronavigation which may impact the consistency of electrode placement and further contribute to variability in results of tDCS studies in aphasia. In the present study, we used neuronavigation guided cathodal tDCS to the right angular gyrus to investigate the impact of tDCS and SLT on scripted conversation and resting-state fMRI and EEG connectivity. Neuronavigation was used at the beginning of every tDCS session to ensure consistent electrode placement. We also performed individual EF modeling to explore whether the direct current was impacting targeted cortical regions, since EF modeling may predict behavioral outcomes [63] and explain variability in the results of tDCS studies [64]. Furthermore, to our knowledge, the present study is the first study in persons with aphasia to use rs–EEG across multiple sessions (i.e., day 1, 5,10, and 15) to explore how functional connectivity changes across the tDCS and SLT treatment sessions.

Results of this study suggest that tDCS in conjunction with SLT improves language performance in persons with aphasia immediately post-treatment. Modest treatment gains were also observed at 4-weeks and 8-weeks post-treatment. These results are consistent with previous studies (e.g., [65,66,67]) that have found a positive impact of tDCS and SLT on behavioral outcomes in persons with aphasia. For example, Fridriksson et al. [22] found that, after three weeks of treatment, performance on trained naming stimuli improved compared to sham tDCS. Moreover, the treatment effects were maintained 4- and 24-weeks post-treatment. Specifically, our study found that cathodal tDCS to the right AG in combination with SLT increased functional connectivity in the left-hemisphere while decreasing functional connectivity in the right hemisphere after 15 treatment sessions. Moreover, the functional connectivity patterns approached those of healthy individuals. These results are in line with Marangolo et al., [25] who found increased resting-state functional connectivity in the left- hemisphere after bilateral tDCS (i.e., anode over the left Broca’s areas and cathode over the right homologue of Broca’s area). Additionally, another case study combining rs–EEG, tDCS, and behavioral measures found that cathodal tDCS to the right-hemisphere contra-lesional area increased activation in the left hemisphere while improving behavioral performance [68]. These results can be explained by the inter-hemispheric inhibition hypothesis, in that suppressing maladaptive right-hemisphere cortical activity can facilitate the recruitment of perilesional areas and induce better language recovery. Results are also in line with models of aphasia recovery, in which perilesional areas far from the lesion are important for language recovery in the chronic phase of aphasia [69]. 

Results also confirm the increase in functional connectivity in DMN after treatment, which can result in positive language changes [70]. The DMN has been shown to have spatial overlap with language processing networks in the frontal and parietal regions and thus are involved in language recovery in individuals with aphasia [71,72]. Although the overall results support the recruitment of perilesional areas for language recovery, we also found that functional connectivity patterns may change throughout the treatment sessions indicating differential effects of treatment on residual brain networks. For example, rs–EEG showed that strong differences in connectivity in the left hemisphere emerged after 15 sessions of treatment rather than 1 or 5 sessions (Figure 7A,B). Therefore, our preliminary results suggest that the number of tDCS and SLT sessions may have an impact on functional connectivity, which in turn may affect behavioral performance. To our knowledge, no prior study has explored how tDCS and SLT may impact functional connectivity across different treatment sessions. Since there is a great deal of variability in tDCS studies of aphasia, we suggest that researchers pay careful attention to the number of treatment sessions that may be required to achieve the beneficial impact of tDCS.

Interestingly, it is noteworthy that the participant’s communication confidence improved post-tDCS and SLT. In his post-treatment interview, P1 also indicated that the treatment of tDCS with SLT improved his conversational performance. Therefore, the impact of tDCS may extend beyond language recovery to psycho-social factors, such as communication confidence, which has been found to be positively correlated with better quality of life [32]. Furthermore, participants’ positive subjective experiences with treatment have shown to have positive psycho-social benefits, which can have a beneficial effect on their broader quality of life [73,74]. However, further research is needed to compare the impact of active tDCS vs. sham tDCS on psycho-social factors, including communication confidence and perceived language recovery, after tDCS and SLT. 

The study has a few limitations. Most notably, we did not include a sham condition and thus it may be difficult to disentangle the specific effects of tDCS and SLT. Therefore, we cannot attribute specific changes in our outcomes to tDCS or SLT alone but rather to tDCS and SLT together. We also did not create an individualized montage for our participant. However, EF modeling in our study showed that direct current was distributed to the targeted brain regions, suggesting that tDCS was administered optimally. 

## 6. Future Directions

Our study lays the groundwork for future multi-modal tDCS and SLT studies. For example, results from our study indicate that rs–EEG changes differentially across different treatment sessions. This may help us in identifying what is the optimal tDCS and SLT dose to achieve improvement in language functioning in persons with aphasia. Another possible direction is to conduct a double-blind randomized controlled study to explore how tDCS and intensive script training improve functional connectivity and behavioral performance more than SLT alone in naturalistic settings. 

## 7. Conclusions

In sum, the study used multi-modal methodology to understand the impact of tDCS and SLT on language recovery in an individual with aphasia. The study used neuronavigation to ensure that electrodes were consistently placed on the brain region across different treatment sessions. Electric field modeling was carried out to simulate the current distribution to the targeted regions, thus ensuring that direct current was impacting the cortical areas we were interested in stimulating. The rs–EEG and rs–fMRI results converged on the impact of tDCS and SLT on increased functional connectivity patterns in the left-hemisphere and its positive impact on language performance beyond single word naming. Therefore, the study provides preliminary evidence from multiple methodologies of how tDCS and SLT may impact brain and behavior relation in aphasia rehabilitation. 

## Figures and Tables

**Table 1:** Participant’s Baseline Western Aphasia Battery-Revised Scores.

Assessment.	Baseline Scores
Western Aphasia Battery-Revised	
Information Content	9/10
Fluency	5/10
Object naming	42/60
Word fluency	9/20
Sentence completion	6/10
Responsive speech	10/10
Yes/no	57/60
Auditory word recognition	41/60
Sequential commands	58/80
Ravens Colored Progressive Matrices	31/36

**Table 2:** Graph measures pre- and post-intervention for P1, and for age- and sex-matched healthy controls.

Measure	Pre-	Post-	Healthy Controls: M, (Bootstrapped 95% CI)
Modularity	0.483	0.544	0.548 (0.527–0.570)
Clustering Coefficient	0.398	0.426	0.466 (0.455–0.478)
Efficiency	0.302	0.293	0.300 (0.283–0.308)
Small Worldness	5.511	5.694	6.296 (6.031–6.570)
Betweenness Centrality	0.007	0.008	0.0099 (0.0094–0.0103)
Participation Coefficient	0.530	0.542	0.514 (0.498–0.531)
Left intra-hemispheric connectivity	0.030	0.041	0.053 (0.051–0.056)
Right intra-hemispheric connectivity	0.101	0.088	0.066 (0.062–0.070)
Inter-hemispheric connectivity	0.031	0.033	0.040 (0.038–0.042)
Lesion volume (cm^3^)	231	231	NA

**Table 3:** Fractional Anisotropy for select white matter tracts.

White Matter Tract	Pre-Intervention	Post-Intervention
All white matter tracts	0.303	0.328
Left Superior Longitudinal Fasciculus	0.238	0.249
Right Superior Longitudinal Fasciculus	0.348	0.375
Left Cingulum	0.380	0.404
Right Cingulum	0.369	0.395

## Data Availability

Data will be made publicly available in the Inter-university Consortium for Political and Social Research (ICPSR) database when the results from a larger clinical trial (NCT03773406) supported by NIDILRR (Grant 90IFRE00020) establishing the efficacy of tDCS in aphasia are published.
